# A prospective, open-label, non-comparative study of palivizumab prophylaxis in children at high risk of serious respiratory syncytial virus disease in the Russian Federation

**DOI:** 10.1186/1756-0500-5-484

**Published:** 2012-09-04

**Authors:** Tatyana V Turti, Elena N Baibarina, Elena A Degtiareva, Elena S Keshishyan, Yurii V Lobzin, Leyla S Namazova-Вaranova, Andrey P Prodeus, Konstantin M Gudkov, Anna I Kruglova, Gregory A Schulz, Gerard F Notario

**Affiliations:** 1 Scientific Center of Children’s Health, RAMS, Lomonosovskiy Prospect, 2/62, Moscow, 119991, Russia; 2 Center for Obstetrics, Gynecology, and Perinatology, Moscow, Russia; 3 Peoples’ Friendship University of Russia, Moscow, Russia; 4 Moscow Science Research Institute of Pediatrics and Pediatric Surgery, Moscow, Russia; 5 Institute for Child Infections, St. Petersburg, Russia; 6 Federal Scientific Clinical Center of Pediatric Hematology, Oncology, and Immunology, Moscow, Russia; 7 Abbott, Moscow, Russia; 8 Abbott, Abbott Park, IL, USA

**Keywords:** Bronchopulmonary dysplasia, Congenital heart disease, Immunoprophylaxis, Lower respiratory tract infection, Preterm infant

## Abstract

**Background:**

Respiratory syncytial virus (RSV) is a leading cause of lower respiratory tract infections (LRTIs) in children globally. Predisposing conditions for the development of serious RSV disease include preterm infants and those with cardiopulmonary illness, including congenital heart disease (CHD) and bronchopulmonary dysplasia (BPD). No vaccine is currently approved for the prevention of RSV infection. It is recommended that children at high risk be prophylactically administered palivizumab, a monoclonal antibody that has been shown in a number of clinical studies to reduce hospitalization rates due to serious RSV infection. The objective of the current study was to determine the safety and effectiveness of palivizumab in preventing serious RSV disease in high-risk children in the Russian Federation. Children at high risk of serious RSV disease (ie, born at ≤35 wk gestational age and ≤6 mo of age, and/or aged ≤24 mo with BPD or hemodynamically significant CHD) were enrolled. Subjects were to receive 3 to 5 monthly injections of palivizumab 15 mg/kg (depending on the month of the initial injection) over the RSV season. The primary endpoint was RSV-related hospitalizations. Adverse events (AEs) were reported through 100 days following the final injection.

**Results:**

One hundred subjects received ≥1 injection of palivizumab; 94 completed their dosing schedule. There were no RSV hospitalizations or deaths. Six of 7 subjects hospitalized for respiratory/cardiac conditions had an RSV test, which was negative in all cases. Three non-serious AEs (acute intermittent rhinitis and rhinitis, 1 subject; atopic dermatitis, 1 subject) were considered possibly related to palivizumab. All other AEs were mild or moderate and considered not related/probably not related to palivizumab.

**Conclusion:**

Palivizumab was generally well tolerated and effectively prevented serious RSV infection in a mixed population of high-risk children in the Russian Federation.

**Trial registration:**

ClinicalTrials.gov: NCT01006629

## Background

Respiratory syncytial virus (RSV) is a leading cause of lower respiratory tract infections (LRTIs) in children globally [1]. In 2005, an estimated 33.8 million cases of RSV-associated LRTIs occurred worldwide in children aged <5 years, with at least 3.4 million resulting in hospitalization and 66,000–199,000 resulting in death [1]. RSV infection is prevalent in the Russian Federation. In a sample of children aged ≤2 years who were hospitalized for LRTI during the RSV season, 38% tested positive for RSV [2]. The prevalence of RSV varied by geographic region and was highest in Moscow (41.7%). Activity peaked in April 2009, when RSV was detected in 62% of children hospitalized for LRTI [2].

Children at high risk for serious RSV disease include preterm infants and those with cardiopulmonary illness, including congenital heart disease (CHD) and bronchopulmonary dysplasia (BPD). The virus infects airway epithelia and manifests as airway obstruction and increased airway resistance [3,4]. Once infection is established, treatment options are limited. Supportive care is the main treatment strategy for serious LRTIs, as there is limited evidence of clinical benefit for the only US Food and Drug Administration − approved antiviral medication for RSV (ribavirin) [5,6]. Further, RSV induces an atypical host immune response that fails to generate robust immunologic memory, leaving patients susceptible to reinfection [3]. Because of the lack of an effective vaccine [7] and the presence of comorbidities, RSV prophylaxis by passive immunization is the primary intervention that can be administered to high-risk children to help prevent serious RSV disease.

Palivizumab (MedImmune, LLC, Gaithersburg, MD, and Abbott, Abbott Park, IL) is a humanized IgG1 monoclonal antibody specific for the F-glycoprotein on the RSV surface [2,8]. It has neutralizing activity in vitro and in vivo, [8] and large clinical trials have demonstrated its efficacy and safety. A large (N = 1502), multicenter, randomized phase 3 trial conducted in the United States, Canada, and the United Kingdom showed that palivizumab prophylaxis reduced RSV-associated hospitalizations in preterm infants and children aged ≤24 months with BPD by 55% compared with placebo (4.8% vs 10.6%, respectively; *P* = 0.00004) [9]. Similarly, in an international study of children aged ≤24 months with hemodynamically significant congenital heart disease (HSCHD; N = 1287), palivizumab prophylaxis resulted in a 45% reduction in RSV hospitalizations compared with placebo (5.3% vs 9.7%, respectively; *P* = 0.003) [10].

Although palivizumab is approved in more than 60 countries worldwide and has been approved for use in the United States and Europe for more than a decade, it only recently (February 2010) received approval in the Russian Federation [2]. This study was initiated before Russian Federation approval and describes the safety and effectiveness of palivizumab in the prevention of serious RSV infection in high-risk children in the Russian Federation during the 2009–2010 RSV season.

## Methods

### Study design

This was a multicenter, prospective, open-label, non-comparative clinical study conducted at 19 sites in 6 geographically diverse cities within the Russian Federation. Eligible participants were children at high risk of serious RSV disease, defined as those born at ≤35 weeks gestational age and ≤6 months of age at enrollment, aged ≤24 months with a clinical diagnosis of BPD (defined as oxygen requirement at a corrected gestational age of 36 wk) requiring intervention or management (ie, oxygen, diuretics, bronchodilators, or corticosteroids) at any time within 6 months before enrollment, and/or aged ≤24 months with HSCHD (cyanotic or acyanotic) either not operated on or only partially corrected. Children with acyanotic cardiac lesions were required to have pulmonary hypertension (≥40 mmHg measured pressure in the pulmonary artery) or the need for daily medication to manage CHD. Exclusion criteria included hospitalization or mechanical ventilation at the time of enrollment, life expectancy <6 months, active respiratory illness or other infection, known renal or hepatic impairment, history of seizures (except neonatal seizures), unstable neurologic disorder, or prior receipt of RSV prophylaxis. A total of 103 subjects were screened; the parents/guardians of 3 subjects refused to sign the informed consent form and these 3 subjects were excluded from the study.

Subjects were to receive palivizumab 15 mg/kg by intramuscular injection every 30 ± 5 days (between 25 and 30 d for the second dose) for a total of 3 to 5 doses over the RSV season, depending on the month of the initial injection. Subjects who developed RSV infection were to continue their dosing schedule, and those undergoing cardiac surgery with cardiopulmonary bypass were to receive an additional injection of palivizumab 15 mg/kg following surgery.

This study was conducted in accordance with the Declaration of Helsinki and complied with Good Clinical Practice according to International Conference of Harmonisation guidelines. The study protocol was reviewed and approved by the Russian Ministry of Health as well as federal and local site ethics committees. Written informed consent from at least 1 parent was obtained for all subjects.

Local site ethics committees that approved this study included OF State Institution Scientific Center of Children’s Health; Children’s Hospital #1 Saint-Petersburg State Institution of Healthcare; Moscow Scientific Research Institute of Pediatrics and Pediatric Surgery; Scientific Research Institute for Children’s Infections; Research Institute of Cardiology, Siberian Branch of the Russian Academy of Medical Sciences; State Institution of Cardiovascular Surgery; Center for Obstetrics, Gynecology, and Perinatology; State Educational Institution of Higher Professional Education, Saint-Petersburg State Pediatric Medical Academy; Scientific Center of Children’s Health of the Russian Academy of Medical Sciences; State Institution of Health; Saint-Petersburg State Institution of Health Care; Kazan State Medical University; Higher Professional Education Novosibirsk Russian State Medical University of Roszdrav; Ivanovo Scientific and Research Institute of Mother and Child named after V.N. Gorodcov Rosmedtechnology; State Educational Establishment of the Highest Vocational Education, Peoples’ Friendship University of Russia; State Educational Institution of Higher Professional Education, Novosibirsk Russian State Medical University of Roszdrav; and Federal Scientific Clinical Center of Pediatric Hematology, Oncology and Immunology.

### Assessments

The primary endpoint was hospitalization due to RSV infection, which included respiratory/cardiac hospitalization with a positive RSV test, development of RSV infection in an already hospitalized subject, or death from RSV infection at any point from the first dose through 30 ± 5 d after the final injection of palivizumab. RSV infection was to be confirmed within 48 hours of hospitalization using an immunochromatographic test for detection of RSV antigen (QuickStripe™ RSV, Savyon® Diagnostics Limited, Ashdod, Israel); nasopharyngeal lavage was the preferred method of specimen collection. Secondary endpoints (assessed through 30 ± 5 d after the final injection of palivizumab) included the number of RSV-associated hospitalization days, number of days with increased supplemental oxygen requirement, intensive care unit (ICU) admissions, number of days in the ICU, and the number of subjects and days of mechanical ventilation, each due to RSV infection. Vital signs, physical examination findings, and medical and social history data were recorded. Safety and tolerability were assessed by treatment-emergent adverse events, defined as events reported from the first dose of palivizumab through 30 days (“+30-day” period) following the final injection, and a second assessment extending to 100 days (“+100-day” period) following the final injection, regardless of suspected causality.

### Statistical analyses

Data were summarized using descriptive statistics. All subjects who received at least 1 dose of palivizumab were included in the analyses.

## Results

**Figure 1:**
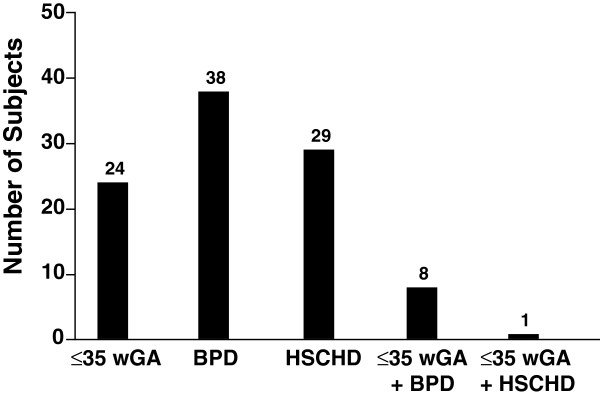
**Number of subjects meeting criteria for high risk of serious RSV disease (N = 100).** BPD = bronchopulmonary dysplasia; HSCHD = hemodynamically significant congenital heart disease; RSV = respiratory syncytial virus; wGA = weeks gestational age.

**Figure 2:**
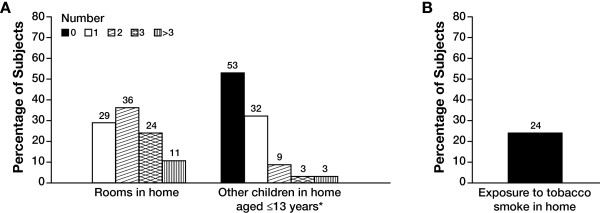
(A) Household conditions and (B) exposure to tobacco smoke (N = 100). *n = 47.

One hundred subjects were enrolled in the study and received at least 1 dose of palivizumab; 94 completed their dosing schedule. One subject discontinued because of an adverse event (a nonserious case of atopic dermatitis) and 5 subjects discontinued because of parent unwillingness to continue participation. A total of 70 subjects were premature and/or had BPD (Figure 1). All subjects were white and the majority (73%) were aged <12 months (Table 1). Mean (SD) gestational age was 33.4 (5.1) weeks and ranged from 24 to 42 weeks, and mean (SD) birth weight was 2.14 (1.02) kg. Mean (SD) age at enrollment was 8.2 (6.3) months and ranged from <1 to 24 months. Concomitant medications were used by 86% of subjects. The most common were vitamins (47%), drugs for obstructive airway disease (38%), systemic antibacterials (22%), diuretics (21%), and cardiac drugs (19%). Household conditions considered risk factors for serious RSV disease or RSV hospitalization are shown in Figure 2. A total of 47% of subjects lived with other young children, 65% lived in a household with only 1 or 2 rooms, and 24% were exposed to tobacco smoke.

**Table 1:** Subject demographic and clinical characteristics (N = 100)

**Characteristic**	
Race,%	
White	100
Gender,%	
Male	48
Female	52
Age at enrollment	
≤6 mo,%	52
<12 mo,%	73
Mean (SD), mo	8.2 (6.3)
Median (range), mo	6.0 (<1 − 24.0)
Mean (SD) birth weight, kg	2.14 (1.02)
Mean (SD) gestational age, wk	33.4 (5.1)
Medical history,%	
Pulmonary*	68
Respiratory distress syndrome/BPD	52
Pneumonia	25
Bronchitis/bronchiolitis	15
Asthma/reactive airway disease	8
RSV-related LRTI	1
Cardiovascular	61
Murmur	42
Pathologic	30
Functional	12
Congenital heart disease	41
Acyanotic	28
Cyanotic	13
Pulmonary hypertension	10
Congestive heart failure	7
Cardiac arrhythmia	1
Hospitalized at birth in intensive/critical care unit,%	67
Hospitalized after birth, prior to study enrollment,%	64

*BPD* bronchopulmonary dysplasia; *LRTI* lower respiratory tract infection; *RSV* respiratory syncytial virus.

*Subjects could contribute to more than 1 category.

All dosing occurred between November 2009 and early April 2010, during the RSV season in the Russian Federation [2]. Most subjects received the last injection in March and thus had coverage in April during the peak of RSV season [2]. Mean (SD) total palivizumab administered per subject throughout the study was 516.5 (192.3) mg. A total of 26% of subjects received 4 doses of palivizumab; 65% received 5 doses. Of the 15 subjects who underwent cardiac surgery, 13 had cardiopulmonary bypass. Four of these subjects received an additional dose of palivizumab (2 of these 4 subjects received a total of 6 doses). Among the remaining 9 subjects, 5 did not receive an additional dose of palivizumab because their regularly scheduled dose was administered shortly after surgery, and 4 had surgery either after study discontinuation (n = 1) or following the RSV prophylaxis period (n = 3).

### Effectiveness

No hospitalizations due to RSV infection occurred during the study. Seven subjects were hospitalized during the study—6 for respiratory and 1 for cardiac conditions. Six of the 7 hospitalized subjects were tested for RSV antigen; all were negative and none were tested by viral culture or PCR-based assay to detect RSV nucleic acids. An RSV test was not performed for 1 subject hospitalized for respiratory reasons; this hospitalization was reported to the investigator several weeks later. The lack of any RSV hospitalizations precluded analysis of secondary endpoints.

### Safety

A total of 80 and 84 adverse events were reported by 41 and 44 subjects in the +30-day and +100-day time periods, respectively (Table 2), most of which were considered not related to palivizumab. Only 3 adverse events (rhinitis and acute intermittent rhinitis [both occurring in 1 subject] and atopic dermatitis) were considered possibly related to palivizumab. The case of atopic dermatitis (considered mild in severity by the investigator) was the only adverse event that led to a subject being discontinued from the study. Adverse events were reported as severe in 3 subjects (arrhythmia, pneumonia, and tonsillitis); however, these events were not considered related to the study drug. Serious adverse events occurred in 10 subjects overall (9 subjects during the +30-day period and 10 during the +100-day period); none were considered related to the study drug. No deaths were reported during the study.

**Table 2:** Most common adverse events* (≥4% of subjects) by severity and relationship to study drug (N = 100)

**Adverse event,% of subjects**^†^	**Total**	**Mild**	**Moderate**	**Severe**	**Related to study drug**
Gastrointestinal disorders^‡^	5	2	3	0	0
Infections	36	20	14	2^||^	1^¶^
Bronchitis	7	0	7	0	0
Rhinitis	19	16	3	0	1^¶^
Upper respiratory tract infection	9	6	3	0	0
Respiratory, thoracic, and mediastinal disorders^§^	4	3	1	0	0
Dermatitis	4	4	0	0	1^¶^

*Adverse events were defined as treatment-emergent events, occurring after study drug initiation and within 100 days after the last dose of study drug.

^†^Subjects could have experienced more than 1 adverse event.

^‡^Includes anal stenosis, enteritis, and teething.

^§^Includes bronchopulmonary dysplasia, rhinorrhea, and tonsillar hypertrophy.

^||^One case each of pneumonia and tonsillitis.

^¶^Possibly related to study drug.

## Discussion

In this study, palivizumab was generally well tolerated and effectively prevented serious RSV infection in a mixed population of high-risk children in the Russian Federation. There were no hospitalizations due to RSV infection, few treatment-related adverse events, no serious treatment-related adverse events, and no deaths reported in this study.

Placebo-controlled clinical trials, [9,10] observational studies, [11,12] and postmarketing data [13-15] from North America and Europe previously established the efficacy of palivizumab prophylaxis. Multiple studies comparing the rates of RSV hospitalization before and after the introduction of palivizumab have demonstrated that RSV hospitalizations were reduced after prophylaxis by 66%–92% in various populations of high-risk children [11,12,15]. One study reported no difference in the rate of RSV hospitalization after the introduction of palivizumab, but the baseline incidence of RSV hospitalization in this population of high-risk children was lower before prophylaxis (3.8%)[16] compared with the baseline RSV incidence for high-risk populations reported in other studies (~13%–44%) [11,12,15]. Postmarketing registry studies conducted in the United States and Canada have also reported low rates of RSV hospitalizations (0.3%–2.9%) in high-risk children who received palivizumab prophylaxis [13,14]. A recent meta-analysis reported that the relative risk of hospitalization for children who had received palivizumab versus placebo was 0.5 (95% CI, 0.38–0.66; *P* < 0.00001) and calculated that the number of patients needed to treat to prevent 1 ICU admission was 20 (95% CI, 9–30) [17]. Consistent with the previously demonstrated effectiveness of palivizumab prophylaxis, there were no hospitalizations for RSV among high-risk children in the Russian Federation in the current study.

Initial clinical trials of palivizumab reported low rates of potentially treatment-related adverse events that were similar to those reported for placebo (7%–11% for both groups) [9,10]. Postmarketing surveillance programs have also shown low rates of adverse events in larger populations. In these studies, serious adverse events were reported in ≤1% of patients [13,14]. This is consistent with the favorable safety profile observed in the current study. There were only 3 adverse events considered possibly related to palivizumab; only 1 of these adverse events (a mild case of atopic dermatitis) led to subject discontinuation from the study.

Limitations of this study include the small sample size and the lack of a placebo control group. Two previous international clinical trials, each enrolling more than 1200 subjects with similar baseline health conditions as in the present study, have shown that the incidence of RSV-associated hospitalizations in subjects who received palivizumab and those who received placebo was ~5% and ~10%, respectively [9,10]. Based on these data, it was expected that several RSV hospitalizations would be observed in the present study with 100 children enrolled. Because the study was conducted during only one RSV season, it is possible that the hospitalization rate may have been lower due to a comparatively mild RSV season. Six of the 7 hospitalized subjects were tested for RSV antigen; all were negative and none were tested by viral culture. The absence of antigen alone does not completely discount the possibility of infection. Nonetheless, the lack of serious RSV infections in this study further supports the overall effectiveness of palivizumab. Formal demonstration of efficacy would have required a control group; however, the results of this study are consistent with the known efficacy and safety profile of palivizumab.

As noted, pivotal clinical trials and postmarketing studies of palivizumab have been conducted in developed, western countries to date. An important consideration in assessing the effectiveness of therapy with palivizumab is the conditions in which high-risk children are living. Environmental and socioeconomic factors, including crowded living conditions, other young children in the home, and exposure to tobacco smoke, may be important risk factors for RSV infection, severe RSV infection, or RSV hospitalization [12,18-21]. In the present study population, nearly half of the subjects lived with other young children, the majority lived in a household with only 1 or 2 rooms, and 24% were exposed to tobacco smoke. Because these factors are often more prevalent in developing countries, [22,23] it was important to conduct this study in a population living under non-western conditions that are representative of much of the developing world.

Premature birth, BPD, and CHD are additional known risk factors for serious RSV disease [19]. The number of children surviving with these conditions in the Russian Federation is expected to increase in 2012, when the country will adopt the World Health Organization definition of live birth. The expanded live birth criteria will include infants born with any sign of life, compared with the current Soviet-era definition requiring respirations, birth weight ≥1000 g, and ≥28 weeks gestational age (unless surviving 7 days) [24,25]. It is anticipated that this change and success in the intensive care of preterm infants with extremely low birth weight in the Russian Federation will increase the number of surviving infants and thus the number considered at high risk for serious RSV disease.

## Conclusions

The current study of palivizumab in a mixed population of children at high risk of serious RSV infection in the Russian Federation is consistent with previous reports of palivizumab safety and efficacy from large, placebo-controlled trials conducted in other countries. These data highlight the importance of access to palivizumab immunoprophylaxis in the Russian Federation.

## Abbreviations

AE: Adverse event; BPD: Bronchopulmonary dysplasia; CHD: Congenital heart disease; HSCHD: Hemodynamically significant congenital heart disease; ICU: Intensive care unit; LRTI: Lower respiratory tract infection; RSV: Respiratory syncytial virus.

## Competing interests

This study was sponsored by Abbott Laboratories. K. Gudkov, A. Kruglova, G. Notario, and G. Schulz are employees of Abbott Laboratories and may hold Abbott stock or options. T. V. Turti, E. N. Baibarina, E. A. Degtiareva, E. S. Keshishyan, Y. V. Lobzin, L. S. Namazova-Вaranova, A. P. Prodeus declare that they have no competing interests.

## Authors’ contributions

TT, KG, GS, and GN conceived of and designed the study. TT, EB, ED, EK, YL, LN, AP, KG, and AK participated in data collection. All authors participated in the design and/or critical revision of the manuscript. All authors read and approved the final manuscript.
